# BRN2 expression increases anoikis resistance in melanoma

**DOI:** 10.1038/s41389-020-00247-1

**Published:** 2020-07-06

**Authors:** Carly J. Pierce, Jacinta L. Simmons, Natasa Broit, Deshapriya Karunarathne, Mei Fong Ng, Glen M. Boyle

**Affiliations:** 1 Cancer Drug Mechanisms Group, Cell and Molecular Biology Department, QIMR Berghofer Medical Research Institute, Brisbane, QLD Australia; 2 Molecular Immunology Group, Immunology Department, QIMR Berghofer Medical Research Institute, Brisbane, QLD Australia; 3 School of Biomedical Sciences, Faculty of Health, Queensland University of Technology, Brisbane, QLD Australia; 4 School of Biomedical Sciences, Faculty of Medicine, University of Queensland, Brisbane, QLD Australia

**Keywords:** Melanoma, Metastasis, Cell biology

## Abstract

Melanoma tumors are highly heterogeneous, comprising of many cell populations that vary in their potential for growth and invasion. Differential transcription factor expression contributes to these phenotypic traits. BRN2, a member of the POU domain family of transcription factors is thought to play important roles in melanoma invasion and metastasis. However, the function of BRN2 during the metastatic process of melanoma remains largely unknown. We therefore investigated the effect of BRN2 expression in melanoma cells with no or low constitutive expression using a doxycycline-inducible system. Induction of BRN2 expression led to reduced proliferation and partial resistance to an inhibitor of mutated BRAF. Whole-genome profiling analysis revealed novel targets and signaling pathway changes related to prevention of cell death induced by detachment from the extracellular matrix, known as anoikis resistance. Further investigation confirmed increased survival of BRN2-expressing cell lines in non-adherent conditions. Functionally, expression of BRN2 promoted induction of c-MET levels as well as increased phosphorylation of STAT3. Treatment with crizotinib, a c-MET inhibitor, decreased cellular viability of BRN2-expressing cells under non-adherent conditions to death by anoikis. Alternative inhibitors of c-MET showed similar results. These results highlight the importance of a largely overlooked transcription factor in the progression and metastasis of melanoma, and may suggest a strategy to target BRN2-expressing cells resistant to therapy and cell death by anoikis.

## Introduction

The incidence of melanoma has increased markedly over the past three decades^1^. It is estimated that ~288,000 people will be diagnosed with malignant melanoma worldwide in 2019, with over 60,000 dying from the disease^2^. The 5-year survival of patients following surgical removal of thin primary tumors (<1 mm thick) is very high. However, metastatic melanoma has historically had an extraordinarily poor survival rate owing to a lack of effective treatments. Although some outstanding progress has been made in treatment of metastatic disease with targeted or immuno-oncology agents, intrinsic or acquired resistance to these therapies remains a serious issue.

Metastatic dissemination of melanoma is a serious complication for the successful treatment of the disease, and represents the most common cause of death for melanoma patients. New strategies to prevent melanoma cell dissemination from primary tumors, and for treatment of metastatic disease are still crucially needed. Metastasis is a complicated multi-step process that can be broadly divided into five stages^3^. Cancer cells initially invade or migrate from the site of the primary tumor into the surrounding tissue, followed by intravasation into the local circulatory/lymphatic system. For metastasis to occur, the disseminated cancer cells must be able to survive in the circulation/lymphatics, before their extravasation from vessels at distant secondary sites. Formation of micrometastatic colonies and proliferation into clinically detectable lesions completes the metastatic process. Metastatic dissemination is a very inefficient process, with only a very small percentage of cells surviving in the circulatory system^4^.

A member of the POU domain family of transcription factors, BRN2 (encoded by the gene *POU3F2*), is thought to play important roles in melanoma formation, progression as well as metastasis. BRN2 is a key regulator of neural crest development, where it controls cell migration^5^. Melanoma cell lines with higher levels of BRN2 are more invasive in vitro^6^. In addition, antisense RNA-mediated ablation of BRN2 results in cells that are slower growing and less able to form colonies in soft agar reflecting a decrease in anchorage-independent proliferation^7^. Previous studies have found that activation of the MAPK pathway in melanoma by mutation of BRAF^8^, nuclear accumulation of β-catenin^9^, PI3K activation^10^, or *CDKN2A* deletion^11^ activate the promoter of BRN2. This leads to the overexpression of BRN2 in melanoma compared with normal melanocytes. BRN2 has recently been shown to suppress apoptosis and reprogram DNA repair, suggesting that BRN2 contributes to generation of high somatic mutation burden in melanoma^12^.

Importantly, BRN2 additionally contributes to melanoma progression through regulation of the master melanocytic transcriptional regulator MITF. BRN2 can either activate^13^ or repress MITF expression^6^ in vitro; however, BRN2 and MITF are present in two distinct sub-populations of cells in 3D culture^14^ and melanoma patient biopsies^6^, where expression of each transcription factor was mutually exclusive. Intra-vital imaging of an engineered mouse melanoma cell line has additionally shown that the motile, invasive cells leaving the site of the primary tumor have high expression of BRN2 while lacking pigmentation markers, suggesting a loss of MITF expression^15^. The relationship between MITF and BRN2 is further complicated, as MITF also has a role in the reduction of BRN2 protein levels^16^. MITF transcriptional activation of a known target gene, *TRPM1* and specifically the microRNA encoded therein (miR-211), results in the reduction of BRN2 protein levels^16^. Our recent work has further highlighted the importance of BRN2 expression, as well as MITF level, in the metastasis of melanoma in vivo^17^.

Most drug-sensitive melanoma cell lines or melanoma patient biopsies have high levels of MITF expression^18^, although overexpression of MITF has been identified as a mechanism underlying resistance to MAPK pathway inhibition^19^. Importantly, low levels of MITF, and presumably high levels of BRN2, have been associated with development of early resistance to targeted therapy^20^. BRN2 has therefore been associated with the invasive, drug-resistant melanoma cell population, whereas MITF with the proliferative, drug-sensitive population. However, it remains largely unclear how BRN2 influences these different cellular phenotypes, and the targets involved.

The current study examined the effects of induction of BRN2 in melanoma cells without constitutive expression. We show that induction of BRN2 expression imparts a slow growth phenotype, and these cells are partially resistant to drug targeting of mutant BRAF. Analysis identified BRN2 as a direct regulator of molecules known to impact resistance to targeted therapy in melanoma, as well as molecules and pathways involved in resistance to anoikis, the cell death induced by cell detachment from extracellular matrix. BRN2 expression was found to lead to enhanced cell viability in non-adherent conditions, characteristic of anoikis resistance. Functionally, induction of BRN2 expression directly increased c-MET levels as well as increased phosphorylation of STAT3, both known to be activated in resistance to anoikis. Pharmacological inhibition of c-MET decreased cellular viability of BRN2-expressing cells under non-adherent conditions, reversing the anoikis resistance phenotype. These results highlight the importance of a largely overlooked transcription factor in the progression and metastasis of melanoma, and show that induction of BRN2 expression leads to cells gaining a drug-resistant phenotype able to survive under non-adherent conditions. BRN2-expressing cells may be targetable with specific inhibitors of downstream targets.

## Results

### BRN2 expression reduces cell proliferation

**Fig. 1:**
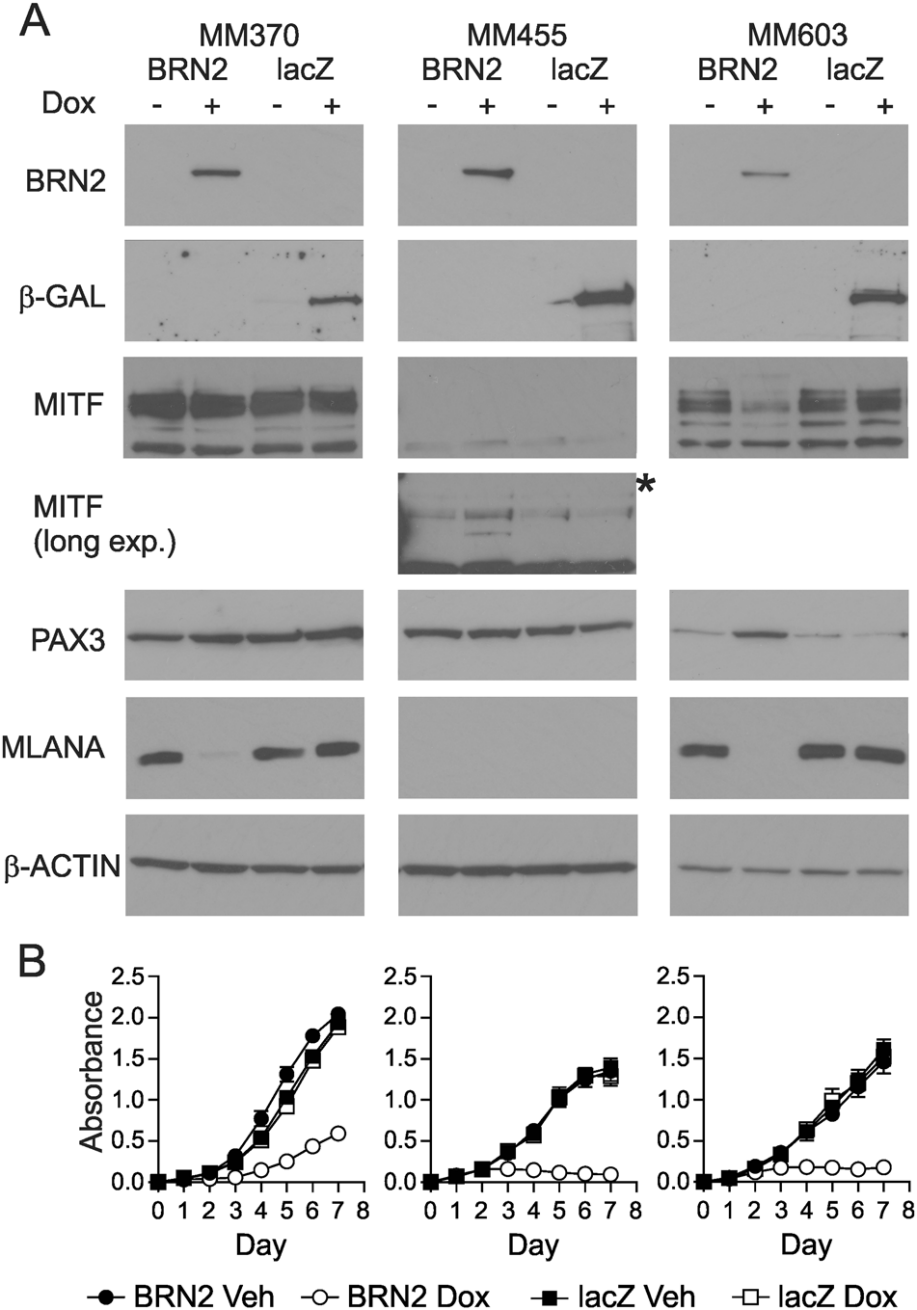
Generation of inducible BRN2-expressing human melanoma cell lines. **a** Production of cells with doxycycline-inducible induction of BRN2 or lacZ in melanoma cell lines. Western blot analysis was performed on MM370, MM455, and MM603 cells, 48 h after induction of expression by exposure to 50 ng/ml doxycycline. * long exposure for MITF in MM455 is also shown. **b** MM370, MM455, or MM603 cells transduced to express BRN2 (circles) or lacZ (squares) were seeded into 96-well plates and treated with doxycycline (open symbols) or vehicle (closed symbols) for 7 days. Sulforhodamine B assay was used to measure cellular proliferation rate. Values indicate mean ± SD; *n* = 3 independent experiments with at least triplicate readings.

To investigate the role of BRN2 in melanoma progression and metastasis, we initially screened a panel of cell lines and determined the constitutive expression of BRN2 (Supplementary Fig. S1). We then sequentially stably transduced melanoma cell lines, including two lines with low levels of BRN2, with lentivirus expressing the tetracycline (Tet) repressor followed by BRN2 or lacZ, as a negative control, under the control of the CMV/TetO_2_ promoter leading to high levels of BRN2 expression (Supplementary Fig. S2). Western blot analysis showed inducible expression of BRN2 or lacZ within 24 hours of doxycycline exposure in all cell lines (Fig. 1a). The three selected lines had varying responses in level of MITF following BRN2 re-expression, specifically slight increase in level (MM455), decreased protein level (MM603) and no apparent change in MITF (MM370; Fig. 1a). PAX3, a transcription factor recently shown to function in a rheostat model with BRN2 to control MITF expression, was detected in all three cell lines^21^ irrespective of MITF level (Fig. 1a). Interestingly, we observed an increase in PAX3 level following BRN2 induction in MM603 cells, where MITF protein level decreased. Knockdown of PAX3 has previously been shown to increase MITF level^22^, suggesting complex interactions between these transcription factors. A previously described MITF target, MLANA, was found to be decreased with induction of BRN2 expression, regardless of change in MITF level in two of the three lines (Fig. 1a). Examination of growth characteristics following induction of BRN2 expression showed marked slowing of cell growth in each line (Fig. 1b), reinforcing that BRN2-expressing cells are slowly proliferating^6,23^. This reduction in proliferation did not correspond to alterations in the level of MITF following BRN2 re-expression (Fig. 1a), and therefore unlikely to be a result of the slow cycling phenotype that was previously observed following a reduction in MITF level^17^.

### BRN2 expression reduces sensitivity to inhibition of MAPK pathway

**Fig. 2:**
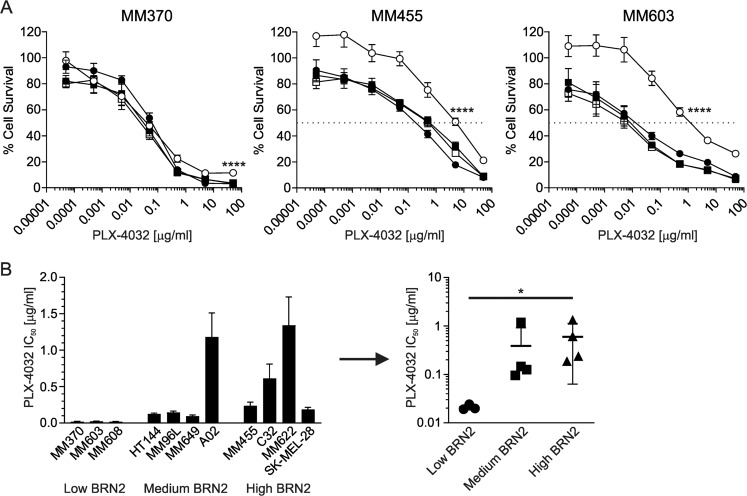
BRN2 expression increases resistance to BRAF inhibition. **a** MM370, MM455, or MM603 cells transduced to express BRN2 (circles) or lacZ (squares) were seeded into 96-well plates, treated with doxycycline (open symbols) or vehicle (closed symbols) the following day before treating with the indicated concentration of PLX-4032 for an additional 5 days. Data were normalized to survival of doxycycline-exposed cells to account for the slower proliferation. Sulforhodamine B assay was used to measure cell survival following exposure to drug. Values indicate mean ± SD; *n* = 4 independent experiment with at least triplicate readings. **b** IC_50_ values of melanoma cell lines with differing expression of BRN2. Left graph shows individual cell line IC_50_ values. Values indicate mean ± SEM; *n* = at least two independent experiments with at least triplicate reading. Right graph shows grouping means for low, intermediate and high levels of BRN2 in cell lines as determined by western blot analysis. Values indicate mean ± SD. *p < 0.05; ****p < 0.0001; unpaired *t* test.

We wished to examine the impact of induction of BRN2 expression on cell survival following exposure to targeted inhibitors of the MAPK pathway, specifically of mutated BRAF (PLX-4032/vemurafenib). Induction of BRN2 expression resulted in significantly increased survival of one of the cell lines at high concentrations of BRAFi (MM370, *p* < 0.0001; *t* test at 50 μg/ml), and overall resistance to BRAFi in two of the lines following BRN2 induction (MM455, *p* < 0.0001; MM603, *p* < 0.0001; IC_50_ values of BRN2 induced with doxycycline versus vehicle following nonlinear regression; Fig. 2a), following normalization of survival to doxycycline-exposed cells to account for the slower cell proliferation. Analysis of sensitivity of cell lines with constitutively high, intermediate or low BRN2 expression further supported a role for BRN2 in mediating sensitivity to BRAFi. Melanoma cells expressing higher levels of BRN2 generally showed higher IC_50_ values compared with those lines with low or no BRN2 expression (Fig. 2b, left). The difference in mean IC_50_ between the high and low BRN2-expressing groups was statistically significant (*p* = 0.0164; Fig. 2b, right). These results suggest that BRN2 expression in part determines responses to BRAFi in melanoma cells, consistent with recently published findings^12^.

### Expression profiling of BRN2-expressing cells reveals uncharacterized targets

**Fig. 3:**
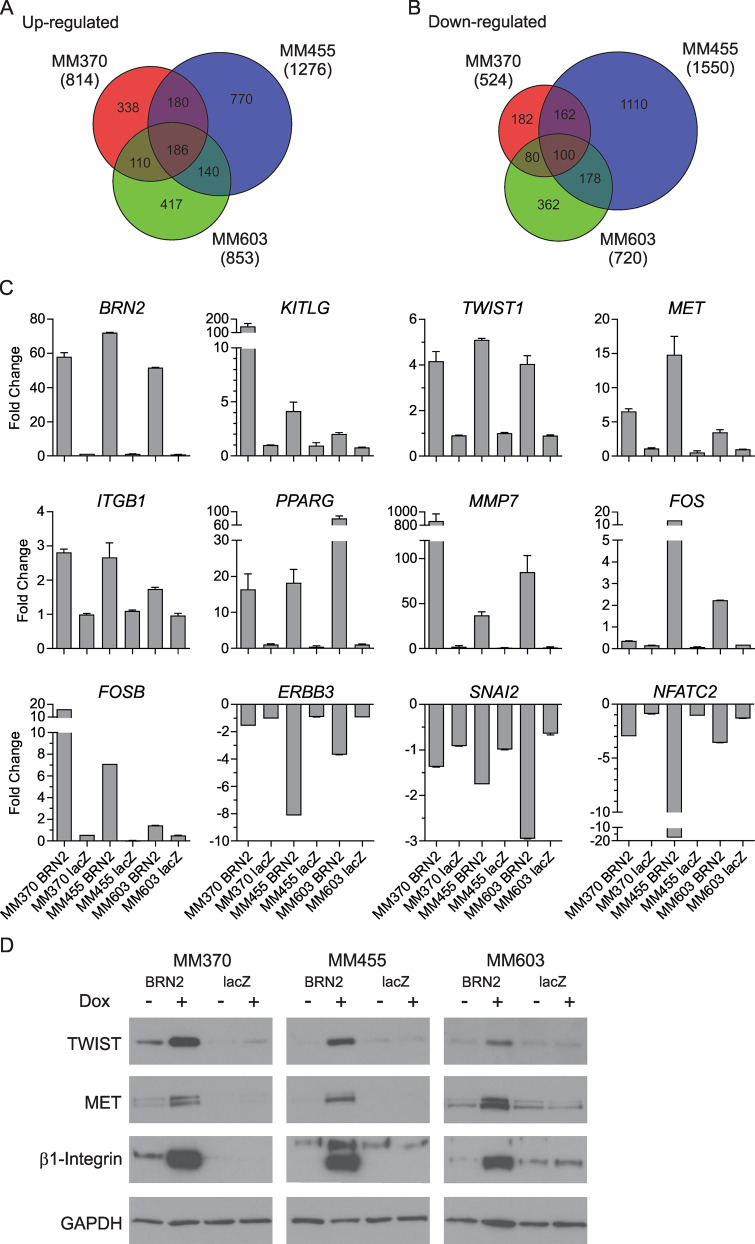
Expression profiling of melanoma cells with induction of BRN2 expression. **a**, **b** Entities at least 1.5 fold **a** up- or **b** downregulated following induction of BRN2 were identified by firstly comparing to the same uninduced cells, then by comparing to lacZ-expressing cells versus uninduced cells to account for changes potentially caused by protein expression or doxycycline exposure. Full data are in Supplementary Tables S1 and S2. **c** qPCR validation of targets following induction of BRN2 or lacZ expression after 48 h exposure to doxycycline. Values indicate mean ± SEM; *n* = at least two independent experiments with at least triplicate reading. **d** Western blot analysis of targets following induction of BRN2 or lacZ expression after 48 h exposure to 50 ng/ml doxycycline or vehicle. Representative blots of two independent experiments are shown.

To understand the consequences of BRN2 expression in melanoma, we used expression profiling to identify differences following induction of BRN2, or lacZ as a control, in all three melanoma cell lines. Total RNA was extracted from BRN2 or lacZ cells treated with doxycycline or vehicle only for 24 or 48 hours before expression profiling analysis (GSE145806). Focusing on changes after 48 hours of induction, we identified BRN2-specific differentially expressed genes (1.5-fold) in at least two of the three cell lines after comparison with lacZ induction. A total of 616 probes from 527 genes were upregulated, and 520 probes from 465 genes downregulated in at least two of three cell lines following BRN2 induction (Fig. 3a, b; Supplementary Tables S1, S2). Previously identified target genes of BRN2, such as *KITLG* and *CD36*^24^, were found to be upregulated in our analysis. We validated a number of genes showing differential expression following induction of BRN2, including *TWIST1*, *MET*, *ITGB1*, *PPARG*, *FOS*, *FOSB*, and *MMP7* as upregulated, and *NFATC2*, *ERBB3*, and *SNAI2* as downregulated in at least two of three melanoma cell lines using qPCR in an independent induction experiment (Fig. 3c). In addition, western blot analysis further validated key changes at the protein level, with TWIST1, c-MET and β1-integrin levels dramatically increasing after induction of BRN2 expression in three of three melanoma cell lines (Fig. 3d).

**Fig. 4:**
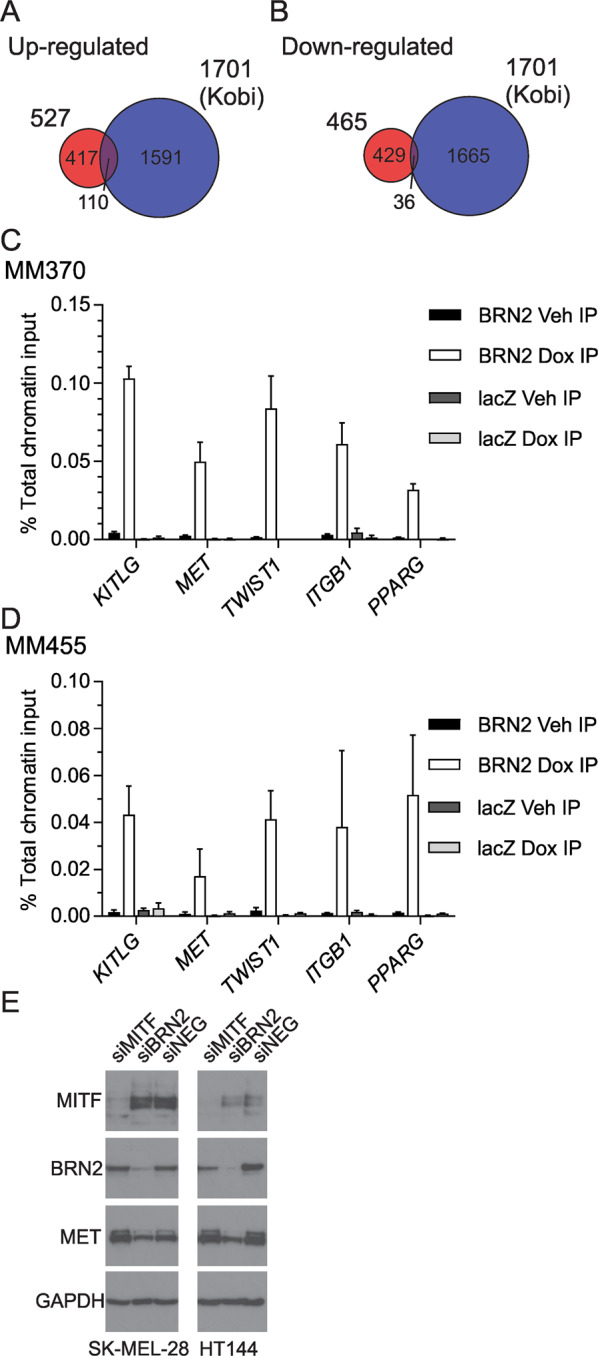
Identification of novel BRN2 target genes. **a** Comparison of upregulated genes following BRN2 re-expression with previously published BRN2 ChIP data^24^. **b** Comparison of downregulated genes following BRN2 re-expression with BRN2 ChIP data. **c**, **d** Chromatin immunoprecipitation (ChIP) assays from **c** MM370 or **d** MM455 cells were performed as described in the Materials and Methods. qPCR was used to determine the percentage of total chromatin input immunoprecipitated by the anti-BRN2 antibody. Values indicate mean ± SD, *n* = triplicate values from three independent experiments. **e** Protein lysates from SK-MEL-28 or HT144 melanoma cell lines treated with 50 nm validated siRNA targeting MITF, BRN2, or GFP as a control (NEG) were examined by western blot analysis. The blots were probed with MITF antibody followed by BRN2, c-MET, and GAPDH antibodies.

We compared our list of differentially expressed genes following induction of BRN2 expression to a list of putative direct BRN2 target genes identified in a previous chromatin immunoprecipitation (ChIP)-seq experiment^24^. The analysis of the two lists showed significant overlap of the upregulated genes with putative targets of BRN2 transcriptional control than what would be expected by chance (Observed 110, expected at *p* < 0.05, 85.05; Chi-squared test, *p* = 0.007; Fig. 4a). The number of downregulated genes was less than what would be expected by chance alone (Fig. 4b). These data suggest some of the targets in the overlap of upregulated genes may be direct transcriptional targets of BRN2 control. We then subjected the gene list of significantly upregulated genes in two of three cell lines following BRN2 induction to pathway analysis using the Ingenuity Pathway Analysis software package. Analysis of the canonical pathways impacted by BRN2 induction identified pathways involved in resistance to anoikis; cell death that is induced upon detachment from extracellular matrix. These included ILK signaling, PDGF signaling, PI3K/Akt signaling, Jak/STAT signaling, ERK/MAPK signaling, and FAK signaling^25,26^ (Table 1, Supplementary Table S3).

**Table 1:** Selected significant canonical pathways altered from induction of BRN2 expression.

Canonical pathway	-log (*p* value)	Ratio	Molecules
ILK signaling	4.53	0.09	ACTA2, FERMT2, FOS, IRS2, ITGB1, NFKB1, PDGFC, PIK3C3, PPP2CA, PPP2R5A, PPP2R5E, PTGS2, RND3, TMSB10/TMSB4X, VCL, VEGFC
PDGF signaling	4.09	0.12	EIF2AK2, FOS, PDGFC, PDGFD, PIK3C3, RALA, RRAS, STAT1, STAT3, SYNJ1
PI3K/AKT signaling	3.81	0.09	CDKN1A, FOXO1, ITGA4, ITGB1, NFKB1, PPP2CA, PPP2R5A, PPP2R5E, PTGS2, RALA, RRAS, SYNJ1
JAK/Stat signaling	3.63	0.11	CDKN1A, FOS, NFKB1, PIK3C3, RALA, RRAS, STAT1, STAT2, STAT3
IGF-1 signaling	3.41	0.10	CCN1, CCN2, FOS, FOXO1, IRS2, PIK3C3, PRKACB, RALA, RRAS, STAT3
CDK5 signaling	3.31	0.09	BDNF, EGR1, FOSB, ITGB1, PPP2CA, PPP2R5A, PPP2R5E, PRKACB, RALA, RRAS
ERK/MAPK signaling	3.29	0.07	FOS, ITGA4, ITGB1, PIK3C3, PLA2G4A, PPARG, PPP2CA, PPP2R5A, PPP2R5E, PRKACB, RALA, RRAS, STAT1, STAT3
HGF signaling	3.21	0.09	CDKN1A, FOS, ITGA4, ITGB1, MET, PIK3C3, PTGS2, RALA, RRAS, STAT3
PAK signaling	3.04	0.09	ITGA4, ITGB1, MYL12B, PDGFC, PDGFD, PIK3C3, RALA, RRAS, WASL
PPAR signaling	2.30	0.08	FOS, NFKB1, PDGFC, PDGFD, PPARG, PTGS2, RALA, RRAS
PTEN signaling	2.23	0.07	CDKN1A, FOXO1, ITGA4, ITGB1, NFKB1, RALA, RRAS, SYNJ1, TGFBR2
mTOR signaling	2.19	0.06	EIF4G3, NAPEPLD, PDGFC, PIK3C3, PPP2CA, PPP2R5A, PPP2R5E, RALA, RND3, RPS6KA3, RRAS, VEGFC
Integrin signaling	2.11	0.06	ACTA2, ARPC5, ITGA4, ITGAV, ITGB1, MYL12B, PIK3C3, RALA, RND3, RRAS, VCL, WASL
AMPK signaling	2.03	0.06	AK3, CDKN1A, FOXO1, IRS2, PIK3C3, PPM1B, PPM1D, PPM1E, PPP2CA, PPP2R5A, PPP2R5E, PRKACB
FAK signaling	1.94	0.07	ACTA2, ITGA4, ITGB1, PIK3C3, RALA, RRAS, VCL

Genes upregulated following 48 h induction of BRN2 expression were analyzed by Ingenuity Pathway Analysis (IPA) software.

### Validation of novel transcriptional targets of BRN2

To validate the identified differentially expressed genes as direct targets of BRN2 control, we performed ChIP analysis using a specific antibody on BRN2 or lacZ-expressing cells. As putative direct targets of BRN2 transcriptional control from the overlap analysis are known to be key regulators of anoikis resistance, we chose to focus on these key molecules likely to impact resistance to anoikis for further investigation and validation. We found BRN2 present at predicted DNA binding sites in the upstream promoter regions of *MET*, *TWIST1*, *ITGB1*, and *PPARG*, as well as the previously described interaction with the promoter region of *KITLG* in both MM370 and MM455 BRN2-expressing melanoma cells compared to cells expressing lacZ, or cells not induced with doxycycline (Fig. 4c, d). One of these validated BRN2 targets, *MET*, has previously been identified as being under the transcriptional control of MITF. To further investigate the relationship, we used transient transfection of siRNA targeting either MITF, BRN2, or lacZ as a negative control. In two different melanoma cell lines (SK-MEL-28 and HT144), we observed that transient knockdown of BRN2 reduced c-MET protein levels, whereas knockdown of MITF had no visible effect on c-MET level (Fig. 4e).

### BRN2 expression increases anoikis resistance

**Fig. 5:**
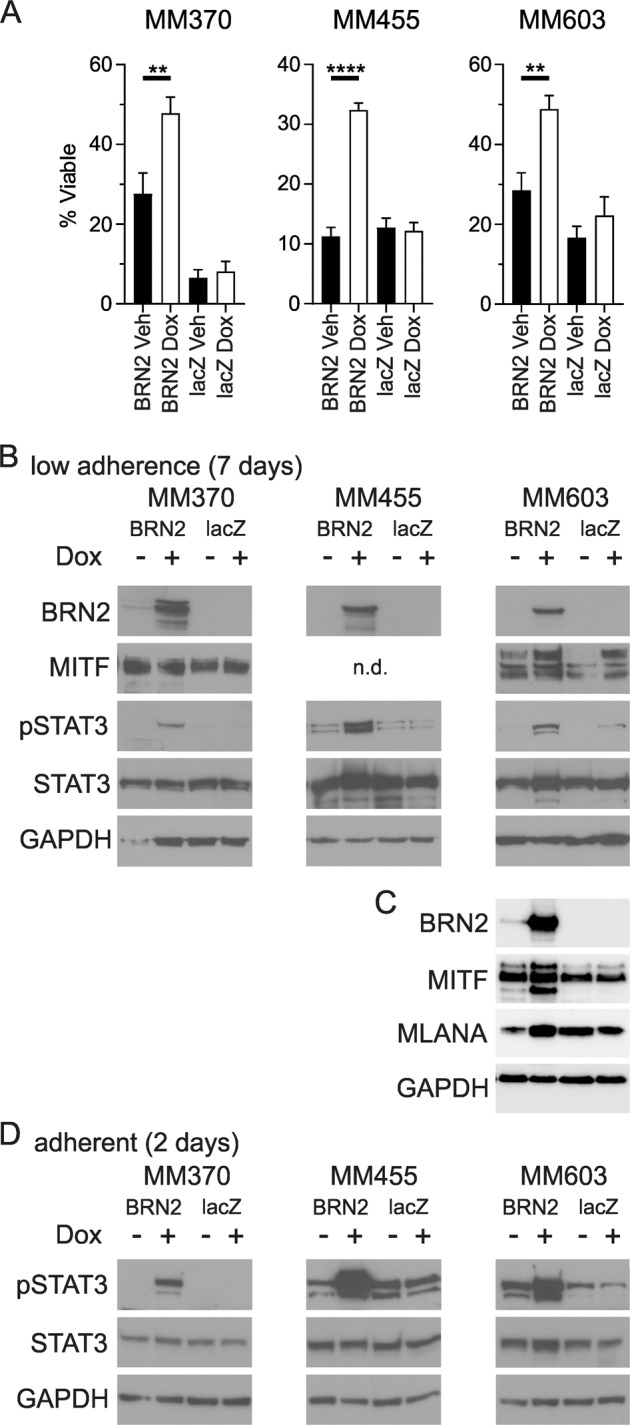
Induction of BRN2 expression induces resistance to anoikis. **a** Cellular viability counts following 7 days of growth under ultra-low-adherence conditions either with or without induction of expression of BRN2 or lacZ as a control. Values indicate mean ± SEM, *n* = at least duplicate counts from three independent experiments. **p < 0.01; ****p < 0.0001; unpaired *t* test. **b**, **c** Protein lysates from melanoma cell lines treated with 50 ng/ml doxycycline or vehicle to induce expression of BRN2 or lacZ as a control for 7 days in ultra-low-adherence conditions were examined by western blot analysis. n.d. – not detected. **d** Protein lysates from melanoma cell lines grown under adherent conditions with BRN2 or lacZ induction for 2 days. The blots were probed with phospho-STAT3 antibody followed by total STAT3, BRN2, MITF (where indicated) and GAPDH antibodies. Representative blots of two independent experiments are shown.

The pathway analysis suggested induction of BRN2 expression altered signaling associated with resistance to anoikis. ChIP assays validated the presence of BRN2 at the promoter sites of key molecules in anoikis resistance. We hypothesized that BRN2 may increase the long-term survival of cells detached from adherent surfaces, characteristic of cells resistant to death induced by anoikis. We therefore assessed cellular viability in ultra-low-adherence conditions, following 7 days of exposure to doxycycline to induce BRN2 expression in three melanoma cell lines. The results showed that induction of BRN2 expression led to significantly increased viability after 7 days in non-adherent conditions, compared with expression of lacZ as a control in all three melanoma cell lines examined (MM370, *p* = 0.09; MM455, *p* < 0.0001; MM603, *p* = 0.0025; Fig. 5a). To understand the molecular mechanism behind the resistance to anoikis induced by BRN2 expression, we revisited the pathway analysis data. The canonical pathways identified as being activated following induction of BRN2 expression included PI3K/Akt signaling, ERK/MAPK signaling and Jak/STAT signaling. In addition, the upstream regulator analysis from IPA identified STAT3 as a likely candidate for the observed gene list (*p* = 9.99 × 10^−18^). Given the previously established importance of activated STAT3 in protection against anoikis in melanoma^27^, we therefore performed western blot analysis of cells with induction of BRN2 expression in low-adherence conditions for a period of 7 days. We found increased levels of phosphorylated STAT3 following induction of BRN2 expression in three of three melanoma cell lines (Fig. 5b). Interestingly, MITF protein level increased in MM603 cells following BRN2 induction under non-adherent conditions (Fig. 5c), in contrast to the changes seen when cells were adherent (Fig. 1a). A downstream target of MITF, MLANA, also showed increased level under non-adherent conditions (Fig. 5c). In addition, we also identified increased levels of phosphorylated STAT3 following 48 h of BRN2 induced expression in cells under adherent conditions (Fig. 5d). These results strongly suggest that BRN2 expression increases the level of phosphorylated STAT3, leading to survival of cells under conditions of detachment and resistance against cell death by anoikis.

### Targeting c-MET resensitizes to anoikis in BRN2-expressing cells

**Fig. 6:**
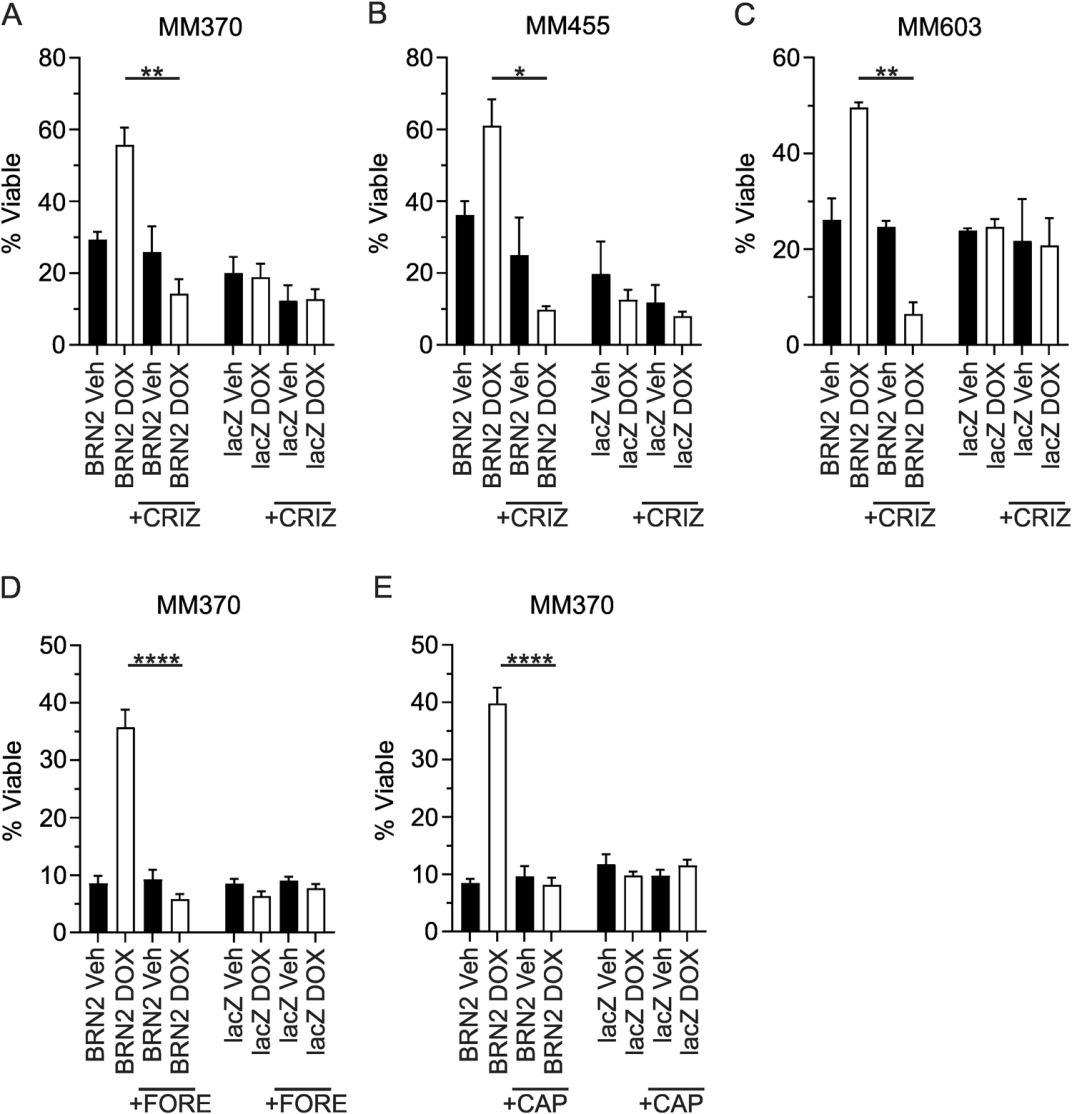
Inhibition of c-MET reverses anoikis resistance induced by BRN2 expression. **a**–**c** Cellular viability counts following 7 days growth under ultra-low-adherence conditions either with or without induction of expression of BRN2 or lacZ as a control with or without inhibition of c-MET with 0.25 µm crizotinib (CRIZ). **a** MM370 cells, **b** MM455 cells, **c** MM603 cells. **d**, **e** MM370 cellular viability counts following 7 days under the above conditions with or without inhibition of c-MET with **d** 0.25 µm foretinib (FORE) or **e** 1.25 nm capmatinib (CAP). **a**–**c** Values indicate mean ± SEM, *n* = at least duplicate counts from three independent experiments. **d**, **e** Values indicate mean ± SEM, *n* = triplicate counts from two independent experiments. *p < 0.05, **p < 0.01, ****p < 0.0001; unpaired *t* test.

Our data suggest that c-MET is a direct transcriptional target of BRN2. Previous data have found that c-MET is a driver of anoikis resistance in cancer^28^, and a well characterized mediator of STAT3 activity^29^. Treatment of melanoma cell lines with hepatocyte growth factor (HGF), the ligand for the c-MET receptor, led to increased levels of phosphorylated STAT3 in two of three parental lines tested (MM455 and MM603; Supplementary Fig. S3a). In addition, BRN2-dependent MET induction was responsive to HGF treatment, as evident by increased downstream STAT3 phosphorylation (Supplementary Fig. S3b). We therefore assessed the potential of pharmacological inhibition of c-MET in reversing anoikis resistance induced by the expression of BRN2 in melanoma. Induction of BRN2 expression in three melanoma cell lines under non-adherent conditions led to increased viability after 7 days. Exposure to 0.25 µm crizotinib, an inhibitor of c-MET, after induction of BRN2 expression led to a significant decrease in cellular viability in non-adherent conditions in all three melanoma cell lines examined (MM370, *p* = 0.0029; MM455, *p* = 0.02; MM603, *p* = 0.0039; Fig. 6a–c). In addition, treatment with crizotinib further decreased cellular viability with induction of BRN2 expression compared with treatment with vehicle only, although this was only significant in MM603 cells (*p* = 0.023). Treatment with additional inhibitors of c-MET, foretinib, and capmatinib, also led to a significant decrease in viability in non-adherent conditions in MM370 cells following BRN2 induction (*p* < 0.0001; Fig. 6d, e). These results suggest the increased cellular viability under non-adherent conditions induced by BRN2 expression is potentially targetable with pharmacological agents against c-MET.

## Discussion

The specific role of BRN2 in the progression and metastasis of melanoma was previously unclear, but has recently drawn very close attention. Pinner et al. demonstrated a potential role for BRN2 in metastasis in a murine melanoma model, with motile and invasive cells exhibiting an active BRN2 promoter region, while lacking pigmentation and therefore potentially MITF expression^15^. Further, antisense ablation of BRN2 in human cells showed a loss of tumorigenic potential, although these cells also had a concomitant loss of MITF expression^7^. Given these data, the question as to whether BRN2 itself contributed to melanoma invasion, or if the effect was solely through the repression of MITF remained largely unanswered. Recent studies have highlighted E2F1 control of BRN2 expression downstream of *CDKN2A* loss^11^, and a role for BRN2-suppressing apoptosis and reprogramming DNA damage repair^12^. In addition, we recently showed that BRN2 may be important for cellular growth following metastatic dissemination in vivo^17^. Taken together, these results have suggested dominant roles for BRN2 in melanoma progression and invasion that are independent of MITF. The data presented here extends these findings by demonstrating BRN2 to have a role in mediating cellular viability under conditions where cells are detached, as occurs following intravasation during the metastatic process. Cell death is usually induced by cell detachment from the extracellular matrix, in a process known as anoikis^25^. Resistance to anoikis is a crucial phenotypic trait that tumor cells must acquire to allow for metastatic spread of disease.

Cells are protected from cell death induced by anoikis when they are in contact with extracellular matrix proteins, via pro-survival and antiapoptotic signals mediated by integrins^25^. Changes in integrin expression therefore have an important role in resistance to anoikis, as does transition from an epithelial phenotype to a mesenchymal state (EMT). Further, activation of several pro-survival pathways have been described as conferring resistance to cell death by anoikis, downstream, or in addition to integrin-mediated signaling. In particular, the ILK and FAK signaling pathways impacting PI3K/Akt, MEK/ERK, and Jak/STAT signaling pathways have all be linked to anoikis resistance of tumor cells^25^. A critical role of STAT3 in anoikis resistance in melanoma^27^ and other tumor types^30^ has been established. The expression profiling analysis of melanoma cells induced to express BRN2 highlighted many of these signaling pathways as being upregulated. Further, our molecular data have additionally demonstrated upregulation of β1-integrin, EMT molecules (TWIST), c-MET, and increased levels of phosphorylated STAT3. We have demonstrated that BRN2 is present in the promoters of, and therefore likely directly controls the expression of these molecules with key roles in anoikis resistance, including c-MET^28,31^, TWIST1^32,33^, and β1-integrin^34^. Recent studies have shown that BRN2 expression protects against cell death through the intrinsic apoptotic pathway^12^. Apoptosis via the intrinsic pathway is the form of cell death resulting from anoikis.

BRN2 induction in adherent cells increased MITF level in MM455, whereas levels were decreased in MM603. Interestingly, although our expression profiling data showed non-significant decreases in MITF mRNA levels for MM370 and MM603, there was no change in MM455 cells. Further, known targets of MITF including *MLANA* and *TYR* (Supplementary Table S2), were also decreased in adherent MM370 and MM603 cells. However, this effect was not recapitulated under low-adherence conditions where BRN2 induction led to increased levels of MITF in MM603 cells, and increases in MITF targets, specifically MLANA. These differences may suggest differential control by the two transcription factors under 2D and non-adherent (3D) conditions. The differences in timing (2 days adherent versus 7 days for low adherence) should also not be dismissed. A weakness of the current study is the non-physiological expression level of BRN2 following induction. However, the elevated levels of BRN2 after 7 days under low-adherence conditions were approaching levels seen in melanoma cells (MM603; Supplementary Fig. S2), with the same phenotypic and expression alterations observed at this timepoint.

It is currently unclear how increased levels of BRN2 lead to high levels of phosphorylated STAT3 within cells. Our analysis, and the previous ChIP analysis of Kobi and colleagues^24^ do not implicate BRN2 in the direct control of STAT3 level, nor the well characterized upstream effectors such as IL6. However, previous work has established that levels of c-MET can impact the phosphorylation of STAT3 (as reviewed in ref. ^29^), and we show that treatment of cells with HGF, the ligand for c-MET, led to an increase in STAT3 phosphorylation in two of three melanoma cell lines (Supplementary Fig. S3a). In addition, the increase in c-MET level following BRN2 induction was responsive to HGF treatment, leading to increased STAT3 phosphorylation (Supplementary Fig. S3b). Importantly, we demonstrate that pharmacological inhibition of the activity of c-MET, a direct target of BRN2, was sufficient to reverse the resistance to cell death by anoikis observed in vitro. This finding adds to recent published data where treatment with the c-MET inhibitor crizotinib strongly inhibited the development of metastasis of uveal melanoma in a mouse model of the disease^35^.

Inhibitors of the MAPK pathway, specifically those targeting mutated BRAF, are known to be more efficacious in rapidly growing cells^36^. Cells induced to express BRN2 are significantly slower growing, potentially impacting the activity of these compounds. In addition, hyperinduction of c-MET expression is thought to be a major driver of resistance to agents targeting activated MAPK in patients with metastatic melanoma^37^. Here, we show that BRN2 expression impacts sensitivity to BRAFi, and also has a role in the direct control of c-MET expression. This last finding is somewhat surprising, given previously published data showing that c-MET is a direct transcriptional target of MITF in melanoma^38^. However, the control of expression is likely complicated and dependent on cellular phenotype, as additional reports have also shown control of c-MET by PAX3^39^.

In summary, these results highlight the importance of BRN2 expression in invasive melanoma cells gaining a drug-resistant phenotype with the potential to survive under non-adherent conditions. Additional clarification of the mechanisms responsible for conferring resistance to anoikis are vital in order to develop strategies to prevent metastasis and treat dissemination of melanoma.

## Materials and methods

### Melanoma cell lines

The cell lines used in this study have been described previously^40,41^. Cells were cultured in RPMI-1640 medium containing 10% heat-inactivated fetal calf serum (Life Technologies, Carlsbad, CA), 100 U/ml penicillin, 100 µg/ml streptomycin, 3 mm HEPES at 5% CO_2_, 99% humidity at 37 °C. Routine mycoplasma tests were performed using PCR and were always negative. Cell line identity was routinely checked by short tandom repeat profiling with the GenePrint 10 System (Promega, Madison, WI) according to the manufacturer’s instructions.

### Production of BRN2-expressing cell lines

The BLOCK-iT Lentiviral Expression System (Life Technologies) was used to produce cell lines stably expressing doxycycline-inducible BRN2 or lacZ (negative control). The sequences were cloned into the pLENTI4/TO/V5-DEST vector. In brief, cell pools were transduced with lentiviral particles containing pLENTI6/TR, and selected with 3 µg/ml blasticidin. Wild-type BRN2 was cloned into pLENTI4/TO/V5-DEST using Gateway recombination. The negative control construct was identical except that it encoded the β-galactosidase gene (Invitrogen, Carlsbad, CA, USA). Lentivirus particles were packaged in 293FT cells, before being titred using MM96L cells. Target melanoma cell lines were transduced with BRN2 or lacZ using a multiplicity of infection of <1, and selected with 500 µg/ml zeocin and 1 µg/ml blasticidin for 3 weeks. Cells were maintained on 100 µg/ml zeocin and 0.5 µg/ml blasticidin for all experiments.

### Proliferation and cell survival assays

To measure cell proliferation, 2.5 × 10^4^ cells were seeded per well into 96-well plates and allowed to attach for 24 h before induction of BRN2 or lacZ expression by the addition of 50 ng/ml doxycycline or vehicle into appropriate wells. Proliferation was determined by fixing plates with methylated spirits each day for 7 days and cell density determined by sulforhodamine B (SRB) assay as previously described^17^. To assess cell survival following drug treatment, cells were seeded at 2.5 × 10^4^ cells per well into 96-well plates and allowed to attach for 24 h. BRN2 or lacZ expression was induced by the addition of 50 ng/ml doxycycline (or vehicle for controls) into appropriate wells. PLX-4032 (SelleckChem, Houston, TX) or dimethyl sulfoxide (DMSO) was then added at the indicated final concentrations and cells allowed to grow for 5 days. Surviving cells were stained using the SRB assay.

### Expression profiling and analysis

Cells were allowed to attach for 24 h before induction of BRN2 or lacZ expression by the addition of 50 ng/ml doxycycline or vehicle for 24 or 48 hours. RNA was extracted using the RNeasy Plus mini kit (Qiagen, Hilden, Germany). Single biotinylated cRNA samples for each cell line treatment and timepoint were prepared with the Illumina TotalPrep RNA Amplification Kit (Ambion, Austin, TX, USA). Labeled cRNA was hybridized to HumanHT-12 v4 BeadChip Arrays (Illumina Inc, San Diego, CA, USA), and then washed and scanned according to standard Illumina protocols. Data were extracted in GenomeStudio (Illumina) using default analysis settings and no normalization method. Resulting data were imported into GeneSpring GX v14.5 (Agilent Technologies, Santa Clara, CA, USA). Expression values were normalized using quantile normalization with default settings. Entities at least 1.5-fold up- or downregulated following induction of BRN2 were identified by comparing with the same, uninduced cells, then by comparing to lacZ expression versus uninduced cells to account for changes caused by protein expression or doxycycline exposure. Significant pathways within the data were assessed using Ingenuity Pathway Analysis (IPA, Qiagen) software. The data discussed in this publication have been deposited in NCBI’s Gene Expression Omnibus, and are accessible through GEO Series accession number GSE145806.

### Quantitative real-time PCR

Total RNA (2 µg) was reverse transcribed with SuperScript III reverse transcriptase (Invitrogen, Carlsbad, CA) following the manufacturer’s protocol. qRT-PCR was performed using the CFX384 Real-Time System (BioRad Laboratories, Hercules, CA) with SYBR Green PCR master mix (Applied Biosystems, Foster City, CA) with the following conditions: 94 °C for 15 min, followed by 40 cycles of 94 °C for 15 s, 60 °C for 15 s, and 65 °C for 3 min, followed by a melt step from 65 °C to 94 °C.). Primers used in this study are listed in Supplementary Table S4.

### Western blot analysis

Western blot analysis has been described previously^42^. Antibodies used in this study were: anti-MITF (12590 S, Cell Signaling Technologies [CST], Danvers, MA), anti-BRN2 antibody (12137 S, CST), anti-MLANA antibody (M7196, Dako, Santa Clara, CA), anti-PAX3 antibody (701147, Invitrogen), anti-phospho-STAT3 antibody (Y705; 91455, CST), anti-STAT3 antibody (9139 S, CST), anti-c-MET antibody (D1C2; 8198 S, CST), anti-b-galactosidase antibody (2372 S, CST), anti-TWIST1 antibody (46702 S, CST), anti-β1-integrin antibody (34971, CST), and anti-GAPDH (R&D Systems, Minneapolis, MN).

### Chromatin immunoprecipitation assays

MM370, MM455, and MM603 melanoma cells, with or without induction of BRN2 or lacZ with 50 ng/ml doxycycline, were grown to 70% confluence. Cells were cross-linked with 1% formaldehyde for 10 min, before addition of glycine solution. The remainder of the ChIP assays were performed using the SimpleChIP Plus Enzymatic Chromatin IP Kit (CST, Danvers, MA) according to the manufacturer’s instructions. Anti-BRN2 antibody (12137 S; CST) was the antibody used for the ChIP assays. Putative binding sites of BRN2 in gene promoters were determined using the SABiosciences database (http://www.sabiosciences.com/chipqpcrsearch.php?app=TFBS). Primers for ChIP analysis are shown in Supplementary Table S4. Real-time PCR products were amplified (RotorGene 6000; Corbett Research, Sydney, Australia) using the SYBR Green PCR Master Mix (Applied Biosystems, Foster City, CA) with the following conditions: 94 °C for 15 min, followed by 40 cycles of 94 °C for 15 s, 60 °C for 15 s and 65 °C for 3 min, followed by a melt step from 65 °C to 94 °C.

### Anoikis resistance assay

MM370, MM455, and MM603 melanoma cells (5 × 10^5^), with or without induction of BRN2 or lacZ with 50 ng/ml doxycycline, were plated into ultra-low-adherence six-well plates (Corning, Lowell, MA). Viable cells were counted after 7 days using trypan blue exclusion, and expressed as a percentage of total cells counted per hemocytometer grid. Crizotinib (PF-02341066), foretinib (GSK1363089), and capmatinib (INCB28060) (SelleckChem) were dissolved in DMSO and used at a final concentration of 0.25 µm (crizotinib and foretinib) or 1.25 nm (capmatinib).

### Statistical analysis

Data is expressed as mean ± error (SD or SEM as indicated). All statistical significance was determined by GraphPad Prism 8.2.1 for Windows (GraphPad Software, San Diego, California USA, www.graphpad.com) using tests as indicated. *p* < 0.05 was considered statistically significant. *p < 0.05, **p < 0.01, ***p < 0.001, ****p < 0.0001.

## Supplementary information

Supplementary Information

Supplementary Information 2

Supplementary Information 3

Supplementary Information 4

Supplementary Information 5

Supplementary Information 6

Supplementary Information 7

Supplementary Information 8

## Data Availability

The data discussed in this publication have been deposited in NCBI’s Gene Expression Omnibus, and are accessible through GEO Series accession number GSE145806.
