# Supplementary material for: BRN2 expression increases anoikis resistance in melanoma
Source: Oncogenesis. 2020 Jul 6;9(7):64. doi: 10.1038/s41389-020-00247-1 (PMC7338542; doi:10.1038/s41389-020-00247-1)
Supplement: Supplementary file 1 — Supplementary Information [file 41389_2020_247_MOESM1_ESM.docx]

**SUPPLEMENTARY FIGURE LEGENDS**

**Supplementary Fig. S1. BRN2 level in melanoma cell lines.** Representative western blot analysis of BRN2 expression in melanoma cell lines used in this study. Western blots were probed with rabbit monoclonal anti-BRN2 antibody followed by GAPDH antibody.

**Supplementary Fig. S2. BRN2 over-expression level in melanoma cell lines.** Representative western blot showing relative level of BRN2 in constitutive BRN2-high expressing cells and cells with BRN2 induction following treatment with doxycycline, either under adherent conditions after 48 hours, or ultra-low adherence conditions after 7 days. Western blots were probed with rabbit monoclonal anti-BRN2 antibody followed by GAPDH antibody.

**Supplementary Fig. S3. HGF treatment increases phosphorylated STAT3 in melanoma cell lines.** **a** Melanoma cell lines were serum starved for 12 hours then treated with vehicle alone or 20 ng/ml hepatocyte growth factor (HGF) for 15 or 30 mins. Western blots were probed with anti-phospho-STAT3 antibody followed by total STAT3 antibody as a loading control. **b** MM370 melanoma cells were treated with vehicle or doxycycline for 36 hours, before being serum starved for 12 hours and treated with 20 ng/ml HGF or vehicle for the final 30 mins where indicated. Western blots were probed with anti-phospho-STAT3 antibody followed by total STAT3 antibody as a loading control.

**SUPPLEMENTARY TABLES**

**Supplementary Table S1.** Up-regulated gene expression following induction of BRN2 with doxycycline for 48 hours. Data from all 3 melanoma cell lines are shown in individual columns. Data was generated by initially comparing to the cells treated with vehicle alone, and then fold change relative to cells expressing lacZ, again doxycycline versus vehicle.

**Supplementary Table S2.** Down-regulated gene expression following induction of BRN2 with doxycycline for 48 hours. Data from all 3 melanoma cell lines are shown in individual columns.

**Supplementary Table S3.** Canonical pathway analysis from IPA following induction of BRN2 with doxycycline for 48 hours.

**Supplementary Table S4.** Primers used in this study.
