# Supplementary material for: BRN2 expression increases anoikis resistance in melanoma
Source: Oncogenesis. 2020 Jul 6;9(7):64. doi: 10.1038/s41389-020-00247-1 (PMC7338542; doi:10.1038/s41389-020-00247-1)
Supplement: Supplementary file 8 — Supplementary Information 8 [file 41389_2020_247_MOESM8_ESM.docx]

**Supplementary Table S4 – Primers used in this study**

***qPCR Primers used in this study***

*POU3F2* F TCATTTCCCCCAATGAATGT

*POU3F2* R AAAGGCCAGTTCCCATACCT

*KITLG* F TCCTGCAGATCCCTTCAGTT

*KITLG* R AGCGCTGCCTTTCCTTATG

*TWIST1* F TCCATTTTCTCCTTCTCTGGAA

*TWIST1* R GTCCGCGTCCCACTAGC

*MET* F TGTTCGATATTCATCACGGC

*MET* R GCATTTTTACGGACCCAATC

*ITGB1* F CTACCAACACGCCCTTCATT

*ITGB1* R ATGTGAATGCCAAAGCGAAG

*PPARG* F CGGAGCTGATCCCAAAGTT

*PPARG* R CCAGAAAGCGATTCCTTCAC

*MMP7* F GCATCTCCTTGAGTTTGGCT

*MMP7* R GAGCTACAGTGGGAACAGGC

*FOS* F CTACCACTCACCCGCAGACT

*FOS* R GTGGGAATGAAGTTGGCACT

*FOSB* F ACCCTCTGCCGAGTCTCAAT

*FOSB* R GAAGGAACCGGGCATTTC

*ERBB3* F GTGCTGGGCTTGCTTTTC

*ERBB3* R TCACACTCAGGCCATTCAGA

*SNAI2* F CAGACCCTGGTTGCTTCAA

*SNAI2* R TGACCTGTCTGCAAATGCTC

*NFATC2* F GGGCCCACTATGAGACAGAA

*NFATC2* R GGCTTGTTTTCCATGTAGCC

*GAPDH* F GGCTCTCCAGAACATCATCCCTGC

*GAPDH* R GGGTGTCGCTGTTGAAGTCAGAGG

***ChIP Primers used in this study***

KITLG ChIP F cacttggaaccatatggatgat

KITLG ChIP R cttggaggtcaaaggttcca

cMET ChIP F tcctcttcaatttcttgcatca

cMET ChIP R tgctaacactgaccaatctgaaa

TWIST1 ChIP F ttgcttgactagcctaaagtcac

TWIST1 ChIP R tgaggcaaaatatttctgagacc

ITGB1 ChIP F ttcactccctttcacccaag

ITGB1 ChIP R cacactgttctcgcctcaaa

PPARG ChIP F gggtgacagagcaagactcc

PPARG ChIP R tcaaaaaggtaactgtgaagtgatg

Neg ChIP F CCTGGAGGGCTTGGAGATG

Neg ChIP R GATCCTACGGCTGGCTGTGA
